# Predicting prognosis in amyotrophic lateral sclerosis: a simple algorithm

**DOI:** 10.1007/s00415-015-7731-6

**Published:** 2015-04-11

**Authors:** Marwa Elamin, Peter Bede, Anna Montuschi, Niall Pender, Adriano Chio, Orla Hardiman

**Affiliations:** Academic Unit of Neurology, Trinity Biomedical Sciences Institute, Trinity College Dublin, Dublin, Ireland; Rita Levi Montalcini Department of Neuroscience, University of Turin, Turin, Italy; Department of Psychology, Beaumont Hospital, Dublin, Ireland; Azienda Ospedaliero Universitaria Cittàdella Salute e della Scienza di Torino, Turin, Italy; Neuroscience Institute of Turin (NIT), Turin, Italy; Department of Neurology, Beaumont Hospital, Dublin, Ireland

**Keywords:** Amyotrophic lateral scleroses, Motor neuron disease, Population-based, Prognoses

## Abstract

The objective of the study was to develop and validate a practical prognostic index for patients with amyotrophic lateral scleroses (ALS) using information available at the first clinical consultation. We interrogated datasets generated from two population-based projects (based in the Republic of Ireland and Italy). The Irish patient cohort was divided into Training and Test sub-cohorts. Kaplan–Meier methods and Cox proportional hazards regression were used to identify significant predictors of prognoses in the Training set. Using a weighted grading system, a prognostic index was derived that separated three risk groups. The validity of index was tested in the Irish Test sub-cohort and externally confirmed in the Italian replication cohort. In the Training sub-cohort (*n* = 117), significant predictors of prognoses were site of disease onset (HR = 1.7, *p* = 0.012); ALSFRS-R slope prior to first evaluation (HR = 2.8, *p* < 0.0001), and executive dysfunction (HR = 2.11, *p* = 0.001). The risk group system generated using these results predicted median survival time in the Training set, the Test set (*n* = 87) and the Italian cohort (*n* = 122) with no overlap of the 95 % CI (*p* < 0.0001). In the validation cohorts, a high-risk classification was associated with a positive predictive value for poor prognosis of 73.3–85.7 % and a negative predictive value (NPV) for good prognosis of 93.3–100 %. Classification into the low-risk group was associated with an NPV for bad prognosis of 100 %. A simple algorithm using variables that can be gathered at first patient encounter, validated in an independent patient series, reliably predicts prognoses in ALS patients.

## Introduction

Amyotrophic lateral scleroses (ALS) is a neurodegenerative disorder characterised by upper and lower motor neuron degeneration and ultimately death from respiratory failure. The rate of disease progression among patients is highly variable [1]. The identification of the key factors that can influence outcome is important for effective timing of medical interventions and for appropriate stratification in clinical trials [2].

Previously reported negative prognostic indicators in ALS include older age of onset, bulbar onset of disease, and short delay to diagnosis [3]. Cognitive impairment, particularly executive dysfunction, has also been shown to be associated with worse prognosis [4, 5]. However, a universally accepted prognostic model that can be utilised in a clinical setting has yet to be established.

The aim of this study was to develop a reliable prognostic model in ALS using information that can be gathered at the first patient encounter, by interrogation of detailed datasets derived from two population-based studies of ALS.

## Methods

The development of the prognostic index and internal validation was carried out in a population-based sample of Irish ALS patients while the external validation of the index was undertaken in a population-based cohort of Italian ALS patients.

The Irish data were generated as part of a large-scale population-based study of cognitive function in incident patients with ALS, performed between October 2006 and February 2011. Details of the population-based Irish ALS Register and study methodology have been previously published [6–8]. In brief, the inclusion criterion was a diagnosis of possible, probable or definite ALS according to the Revised El Escorial criteria [9]. Exclusion criteria were confined to conditions that could affect neuropsychological function, such as major hemispheric stroke or alcohol dependence syndrome. All clinical and neuropsychological data were gathered during home-visits and patients were followed prospectively from diagnosis to time of death.

The Italian cohort comprised a sample of incident patients (*n* = 122) from a population-based study of cognition undertaken in the provinces of Torino and Cuneo of Piemonte region [10]. All patients were diagnosed between 1 January 2009 and 31 December 2011 with definite, probable and probable laboratory-supported ALS according to the revised El Escorial and were identified through the Piemonte and Valle d’Aosta register for ALS [11]. Exclusion criteria, previously published, were neurological conditions that can affect cognition [10].

Disease severity in both studies was estimated using the Revised ALS Functional Rating Scale (ALSFRS-R) [12]. A retrospective estimate of the average rate of functional decline prior to time of first evaluation was computed by dividing the difference between the ALSFRS-R scores obtained by the patient and a presumed normal score (48 points) at symptom onset by disease duration (in months) at time of evaluation. This measure is termed the ALSFRS-R-based linear estimate of rate of disease progression [13]. For the sake of simplicity we will refer to it from here onward as the ALSFRS-R slope.

All patients underwent comprehensive neuropsychological assessments [6, 10]. Three executive tasks were selected from each database to evaluate executive dysfunction. The choice of tasks was based on the available literature, including the Irish dataset, regarding the tasks’ sensitivity to executive dysfunction in this patient population. Tasks used to evaluate executive function in the Irish cohort were the Stroop Colour–Word Interference task [14], verbal fluency (phonemic verbal fluency index and semantic fluency) [15], and the backward digit span. Normative data were generated using a large cohort (*n* = 136) of age, sex and education-matched healthy controls. Tasks used to evaluate executive function in the Italian cohort were the Stroop Interference Colour–Word task [14], verbal fluency (FAS phonemic fluency) and the Trail making A and B test and normative data were generated using Italian age, sex, and education-matched controls (*n* = 127). In both cohorts, executive dysfunction was defined as a score that is two standard deviations below that of the corresponding control mean on at least two of the three selected tasks.

Patients with established ALS-causing mutations were identified using either targeted next-generation sequencing, or repeat-primed PCR [16, 17].

### Statistical analysis

To formulate and test the prognostic index, a three-stage process was carried out.

The Irish cohort was split randomly into two sub-cohorts: a Training and a Test sub-cohort comprising approximately 60 and 40 % of the cohort, respectively. Baseline characteristics of the two sub-cohorts were compared using two-sample *t* test or Mann–Whitney *U* test depending on whether the variable displayed normal distribution or not. The Chi-square test was used for comparing proportions, with Monte Carlo correction where appropriate.

Data from the Training sub-cohort were used to identify significant predictors of prognosis and generate the prognostic index and prognostic classification system. Survival time (in months) was computed from date of symptom onset to time of death from all causes. Potential predictors were selected based on the available literature and the likelihood of availability at first clinical assessment. Variables that had a significant effect on survival on univariate analyses were included in multivariate analyses. In the case of categorical variables, univariate analyses were carried out using Kaplan–Meier survival methods and the log-rank test was used to test equality of outcome. Cox proportional hazards regression analyses were undertaken in case of continuous variables and for building multivariate models (after ensuring that the assumption of proportional hazards was not violated). Patients who were alive at the time of analysis were censored. After identifying significant predictors of prognosis on multivariate analyses, internal validation of the model was carried out using boot-strapping techniques using 1000 random samples to obtain 95 % confidence.

Based on the results of the survival analyses, the prognostic index was generated by assigning weighted scores to each factor (higher scores for worse prognoses) guided by the hazard ratio (HR) suggested by the multivariate Cox proportional model. Continuous variables with significant survival effects on both univariate and multivariate analysis were converted to categorical variables to allow easier formulation of the prognostic index. Patients were then classified into risk groups based on total index score, with higher scores predicted to be associated with worse outcome.

Lastly, the classification system was tested in the Irish Test sub-cohort (internal validation) and the Italian cohort (external validation). This was carried out using Kaplan–Meier method estimated median survival times and by comparing the proportion of patients from each prognostic risk group who had either (1) poor prognosis, defined as survival time of 25 months or less from symptom onset or (2) good prognosis, defined as survival time of at least 50 months or more from symptom onset.

All tests were two-tailed and statistical significance was set at *p* < 0.05. Statistical analyses were carried out using SPSS version 21 (SPSS Inc. Chicago, Illinois).

Written informed consent was obtained from all participants. The Irish study has full ethical approval from Beaumont Hospital Research Ethics Committee while the Italian study has full ethical approval from the San Giovanni Hospital of Turin Ethics Committee.

## Results

The Irish population-based cohort represented 61.6 % of patients diagnosed with ALS in the Republic of Ireland during the set study period (244/396). Forty patients were subsequently excluded, and the remaining 204 patients were included in the final study. Reasons for non-capture included death prior to contact (*n* = 95) and patients declining participation (*n* = 47) or not responding to invitation (*n* = 10). Reasons for exclusion included history of major hemispheric stroke (*n* = 9), alcohol dependence syndrome (*n* = 6), pre-morbid learning disability (*n* = 1), major psychiatric disorder (*n* = 3), atypical disease course suggestive of variant (*n* = 4), severe active epilepsy (*n* = 1), patients being too sick to participate adequately in the study (*n* = 12), patient not fully informed of diagnosis (*n* = 1) and co-morbid Alzheimer’s disease at baseline (*n* = 3).

Recruited ALS patients displayed no significant differences with regard to age, sex distribution, or site of onset when compared to patients who were diagnosed in the same period but did not participate in the study, although non-participants experienced a shorter median survival time (*p* < 0.0001).

At time of analysis (May 2014), 177 of the 204 patients in the Irish cohort were deceased (86.8 %). Median survival time from symptom onset of the deceased patients was 32 months (range 7–126, interquartile range = 21). Among patients who remained alive (*n* = 27) the median follow-up time was 47 months from study enrolment, and median follow-up time measured from symptom onset was 75 months (range 51–114).

There were no significant differences in baseline characteristic between the two Irish sub-cohorts (Training and Test groups, see Table 1).

**Table 1:** Baseline characteristics of the two Irish sub-cohorts (the training and test sets) and the Italian validation cohort

	Irish cohort		*p* value	Italian cohort
	Training set	Testing set		
*N*	117	87		122
Mean age at symptom onset (SD)	60.8 (10.3)	62.7 (10.4)	0.351	65.6 (10.5)
Males	61.5 %	55.2 %	0.441	58.2 %
Mean education in years (SD)	12.0 (3.4)	12.3 (3.0)	0.528	9.1 (4.2)
Site of onset				
Spinal	65.5 %	57.0 %	0.353	63.9 %
Bulbar	33.6 %	40.7 %		36.1 %
Respiratory	0.9 %	2.3 %		0.0 %
Median delay to diagnosis (months)	10.0	12.0	0.444	8
Median disease duration (months)	17.0	19.0	0.652	10
Mean ALSFRS-R (SD)	35.7 (7.9)	37.1 (7.0)	0.194	39.8 (5.8)
FH of ALS	12.0 %	13.8 %	0.861	8.1 %

Data from the Training sub-cohort were used to identify significant predictors of survival time. Univariate survival analysis was carried out for the following clinical variables: age at symptom onset, gender, site of disease onset (spinal-onset versus non-spinal or bulbar/respiratory onset), diagnostic category as per the El Escorial (possible, probable or definite), the ALSFRS-R slope (48-ALSFRS-R/disease duration at time assessment), the presence (versus absence) of family history of ALS and/or frontotemporal lobar degeneration in a 1st or 2nd degree relative, and the presence (versus absence) of executive dysfunction on cognitive testing.

Factors associated with significantly worse prognosis on univariate analyses in the Training sub-cohort (*n* = 117) were (1) Bulbar or respiratory (i.e. non-spinal) onset of disease with a median time of 30 months (95 % CI 26.9–33.1, SE 1.6) compared to 36 months in patients with spinal-onset disease (95 % CI 30.9–41.4, SE 2.6, *p* = 0.032); (2) higher ALSFRS-R slope (indicating faster functional decline), HR 2.6, 95 % CI 1.9–3.5, SE 0.15, *p* < 0.0001; (3) and the presence of executive dysfunction (median survival = 27 months, 95 % CI 19.9–34.1, SE 3.6) as opposed to absence of executive dysfunction (median survival time 37 months, 95 % CI 28.2–45.8, SE = 4.5, *p* < 0.000).

Although patients with older age at symptom onset and female patients tended to have shorter survival, the effect did not reach statistical significance in either case (*p* = 0.094 and *p* = 0.064, respectively). Similarly, a positive family history for ALS and/or FTLD and El Escorial diagnostic category at diagnosis had no significant effect on survival on univariate analyses (*p* = 0.972 and *p* = 0.109, respectively).

Proportional hazards Cox regression was used to build a multivariate model that included site of disease onset, ALSFRS-R slope, and executive dysfunction (*n* = 117). The survival effect of all the three factors persisted on multivariate analyses: (1) non-spinal onset of disease, HR = 1.7 (95 % CI 1.12–2.63, SE 0.22, *p* = 0.012); (2) ALSFRS-R slope: HR = 2.8 (95 % 2.00–3.81, SE = 0.166, *p* < 0.0001); and executive dysfunction: HR = 2.11 (95 % 1.37–3.28, SE = 0.233, *p* = 0.001). Internal validation of the model was carried out using boot-strapping techniques. Based on the results of 1000 randomly generated samples, the robustness of the three-parameter model was confirmed.

**Fig. 1:**
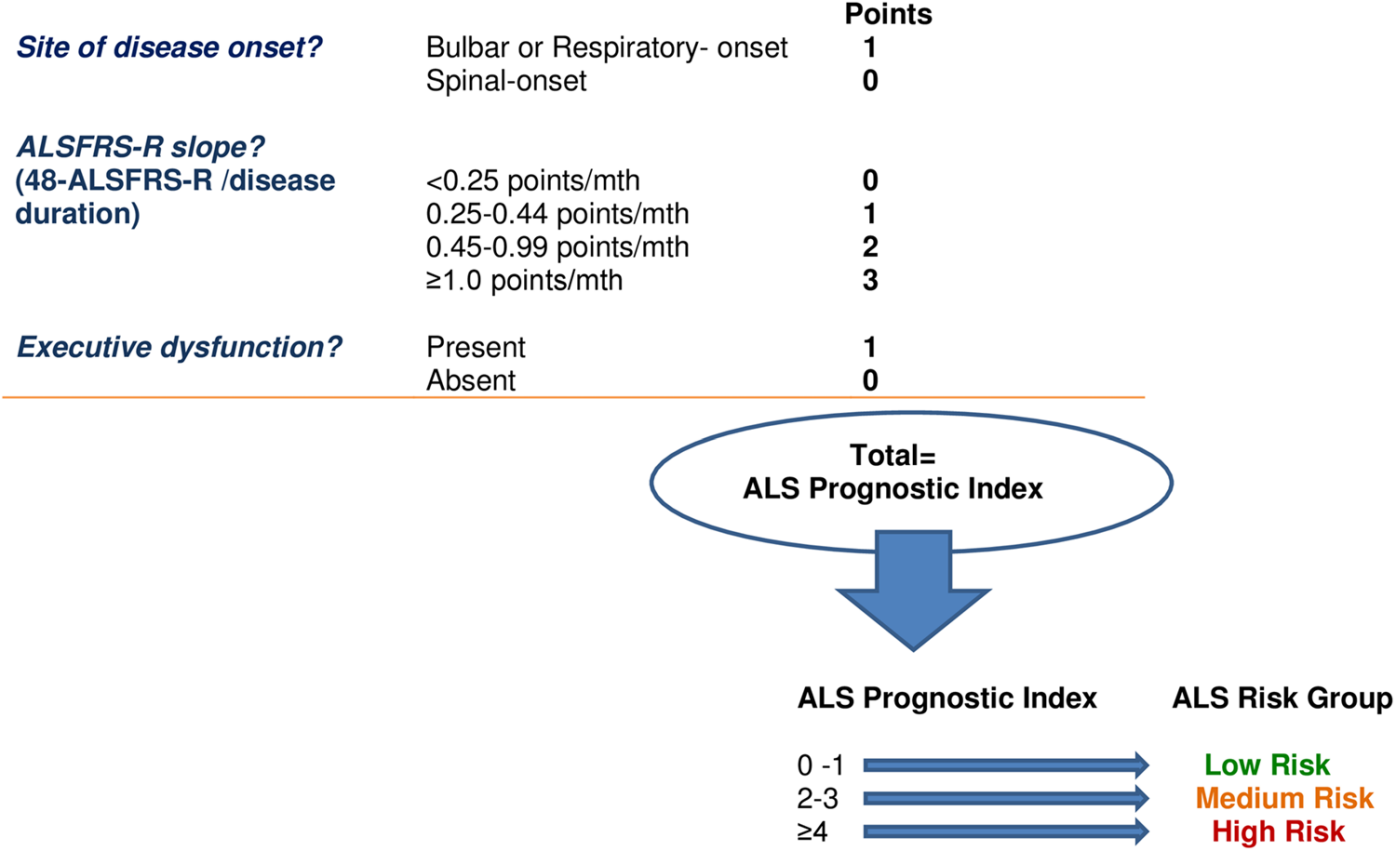
This figure illustrates how to calculate of the ALS Prognostic Index for individual patients and how to allocate patients to the ALS risk groups

Based on these results a simple prognostic index, named the ALS Prognostic Index (or API), was generated (Fig. 1) with possible scores ranging from zero to six (higher scores indicating worse predicted prognosis). The figure also shows how patients were then divided using the total API score into three risk groups, termed the ALS risk groups.

The index and prognostic risk group classification procedure were applied to the Irish Training set. The index and classification were also applied to the Irish Test sub-cohort (after excluding one patient for missing data precluding full classification, *n* = 86) and, for external validation purposes, to the Italian cohort (*n* = 122).

**Fig. 2:**
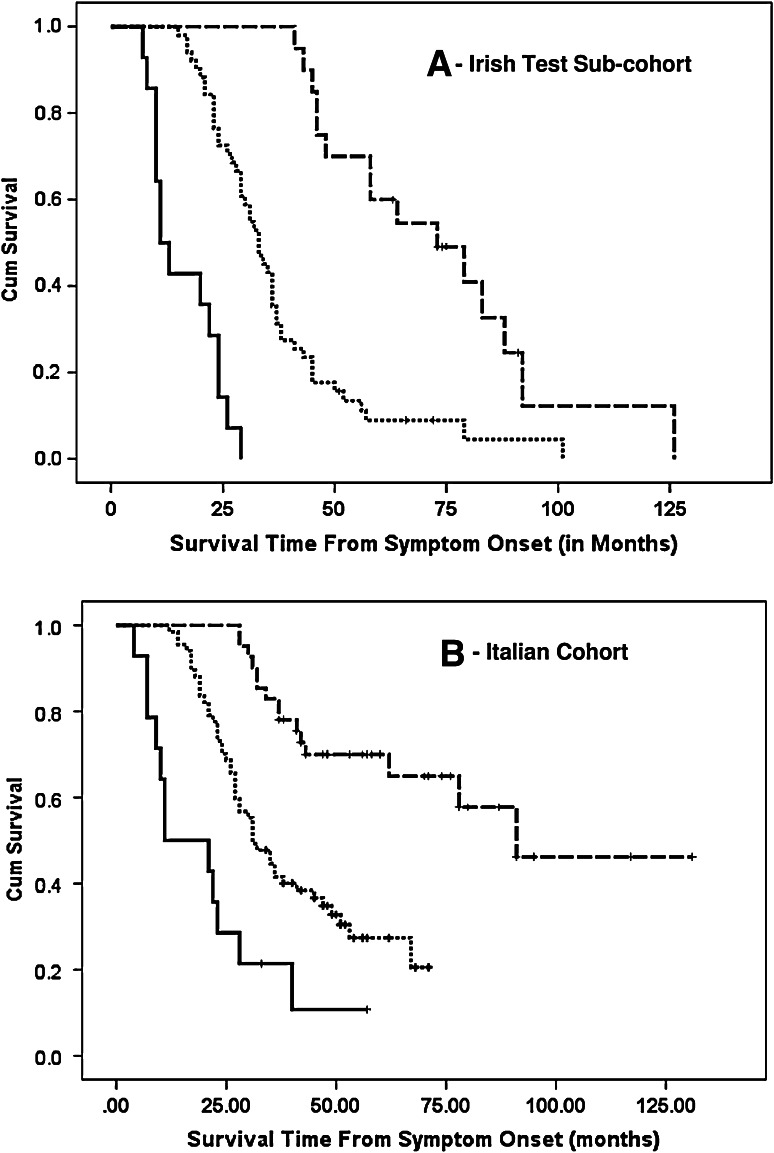
Figure shows Kaplan–Meier plots for survival probabilities in the Irish test cohort (**a**) and Italian cohort (**b**). In all cases ALS patients were stratified by ALS prognostic risk group. *Dashed line* low-risk group, *dotted line* medium-risk group, and *solid line* high-risk group

As shown in Table 2, in all three cohorts the ALS risk groups predicted survival time (log-rank test *p* < 0.0001 in all three cohorts) with no overlap of the 95 % confidence intervals (Kaplan–Meier survival plots for validation cohorts shown in Fig. 2).

**Table 2:** This table summarises the Kaplan–Meier estimated median survival time for the three ALS risk groups in the Irish training and test cohorts and the Italian cohort

	High-risk group			Medium-risk group			Low-risk group			Log-rank *p* value
	*N*	Median survival time	95 % CI	*N*	Median survival time	95 % CI	*N*	Median survival time	95 % CI	
Training set	26	22.0	16.0–28.0	64	34.0	30.5–37.5	27	63.0	50.1–75.9	<0.0001
Irish test set	14	11.0	7.3–14.7	51	33.0	28.6–37.4	21	73.0	48.0–98.0	<0.0001
Italian cohort	18	22.0	19.2–24.8	72	35.0	26.9–43.1	32	91.0	63.6–118.4	<0.0001

**Fig. 3:**
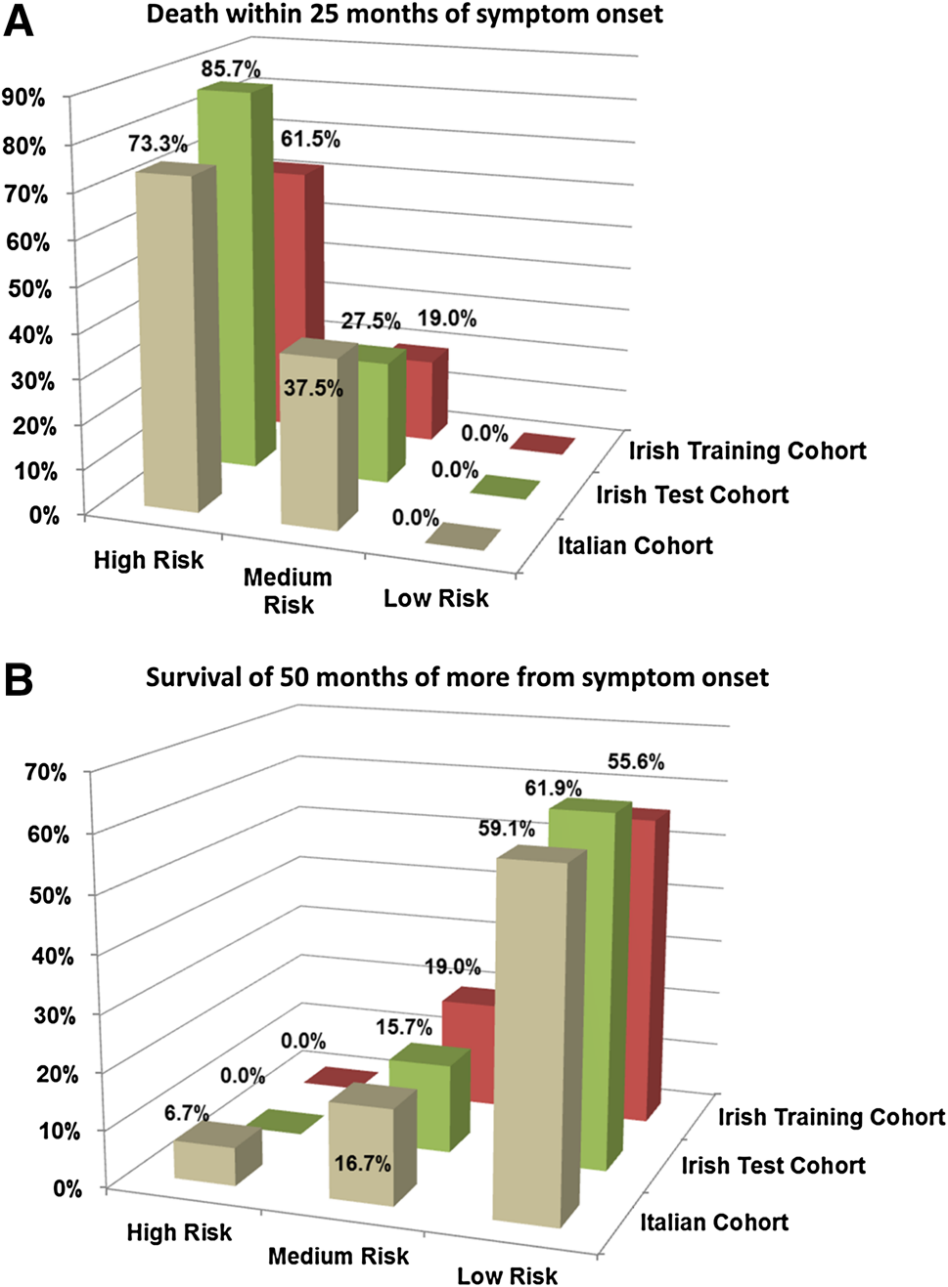
This figure illustrates proportion of patients in each cohort stratified by API risk group who **a** died within 25 months of symptom and **b** had a survival time of 50 months or more

To investigate the utility of the ALS risk group classification in predicting risk of (1) poor prognosis, defined as death within 25 months of symptom onset and (2) good prognosis, defined as survival time of at least 50 months post-symptom onset, we included only patients who were either deceased at time of analyses or whose follow-up time measured from symptom onset was at least 50 months (all Irish patients and 91 Italian patients). In all three cohorts, the API risk group was a reliable predictor of both poor and good prognosis (Fig. 3a, b, Chi-square test *p* < 0.0001 in all cases). In the validation cohorts, classifying a patient into a high-risk group was associated with a positive predictive value for poor prognosis of 73.3–85.7 % and a negative predictive value for having good prognosis was 93.3–100 %. Conversely, the low-risk group was associated with a positive predictive value for good prognosis of 59.1–60.1 % and negative predictive value for bad prognosis of 100 %.

**Fig. 4:**
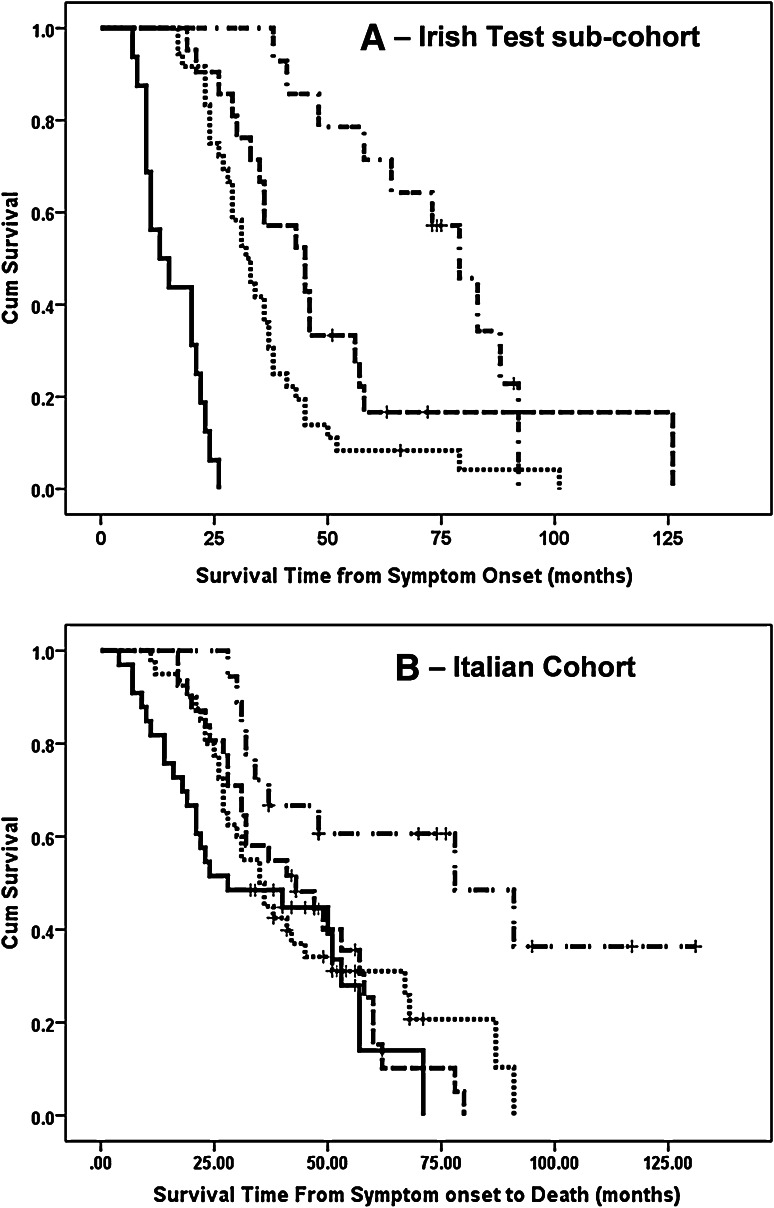
Figure shows Kaplan–Meier plots for survival probabilities in the Irish test cohort (**a**) and Italian cohort (**b**). In all cases ALS patients were stratified by ALSFRS-R slope only. *Solid line* ALSFRS-R slope of 1.0 points/month or more, *dotted line* ALSFRS-R slope 0.50–0.99 points/month, *dashed line* ALSFRS-R slope 0.25–0.49 points/month, *dash and dot line* ALSFRS-R slope <0.25 points/month

As the ALSFRS-R slope was the strongest predictor of survival in the model, we investigated the utility of a classification system based on this measure only (ALSFRS-R slope <0.025 points/month, 0.25–0.49 points/month, 0.50–0.99 points/month, and ≥1 points/months). As shown in Table 3 and Fig. 4, although this model was useful in the Irish validation cohort with only minor overlap of survival times, it was poor predictor of survival in the Italian cohort (external validation cohort).

**Table 3:** This table summarises the Kaplan–Meier estimated median survival time for the patients in the Irish test cohorts and the Italian cohort classified by ALSFRS-R slope only

ALSFRS-R slope (points/month)	Irish test set			Italian cohort		
	*N*	Median survival	95 % CI	*N*	Median survival	95 % CI
<0.25	14	79.0	66.4–91.6	18	78.0	28.3–127.7
0.25–0.45	21	45.0	31.6–58.3	31	43.0	23.5–63.5
0.50–0.99	36	36.0	27.3–36.7	40	35.0	27.3–42.7
≥1.0	16	16.0	27.3–36.7	33	28.0	4.1–51.5
Log rank *p* value	<0.0001			0.004

### Risk groups and genetic status

We investigated the relationship of the risk group allocation and the *C9orf72* pathogenic hexanucleotide repeat in both populations. Genetic screening for common ALS genetic mutation was undertaken in 197 Irish patients (96.6 % of the cohort). *TARDP* gene and *FUS* gene mutations were identified in one patient each (0.5 % of cohort in each case) and 19 patients carried the *C9orf72* hexanucleotide repeat expansion (9.3 %).

Carriers of the *C9orf72* repeat expansion represented 10.6 % of the medium-risk group and 13.2 % of the high-risk groups compared to 4.4 % of the low-risk group, although the difference did not reach statistical significance.

In the Italian cohort genetic status was available in all 122 cases, with *TARDP* gene mutation identified in 5 cases, *FUS* and *optineurin* in 1 case each. Three patients carried the *C9orf72* repeat expansion (2.6 %) with two cases in the Medium Risk and 1 case in the High risk, representing 2.8 and 5.6 % of each group, respectively, and no *C9orf72* positive cases in the low-risk group.

## Discussion

Heterogeneity of disease progression in ALS is a major confounder of clinical trials, and a validated, reliable and practical prognostic model for ALS patients is urgently required [18]. Accurate prognostic stratification also has pragmatic implications for the management of individual patients, such as feeding tube placement, end-of-life decisions, putting supportive services in place, timely referral for palliative care [19]. However, a reliable model that can be used in a clinical setting model has remained elusive. Proposed models to date have been excessively complex to allow practical use in the setting of a busy clinic or a clinical trial centre and, to our knowledge none has been validated using more than one population [20–24].

This study has utilised data-led hypothesis-free analyses of prospectively gathered population-based data to generate a simple predictive model. The objective was to formulate a model using clinical and neuropsychological data that can be gathered at first evaluation of the patient that can reliably identify those with poor prognosis. External validation was carried out using independently gathered data from a different population.

Our data support the utility of retrospective computation of the rate functional motor decline prior to first evaluation an estimate of disease progression rates in individual patients [13]. Similarly, the association of bulbar and respiratory onset of disease with poorer prognosis has also been previously described [3, 25, 26], and probably reflects the earlier presentation of swallowing and respiratory difficulties in these patients.

Our study now incorporates for the first time cognitive status into a prognostic model. We and others have already shown that executive dysfunction is predictive poor prognosis [5, 10, 27]. However, full assessment of cognitive status remains a challenge. We have estimated that the total time required to perform the tasks included in this study is approximately 30–40 min. As standardised clinic-based screening tools to assess cognitive and behavioural status are now available [28, 29], it would be desirable to replicate our findings using these tools.

Previously published prognostic models have reported conflicting data regarding the reliability of prognostic factors such age, gender, El Escorial diagnostic category [1, 3, 20, 21, 26, 30–32]. Environmental factors such as smoking, socio-economic status, marital status, and multidisciplinary care have also been reported to affect prognosis in ALS [26, 32, 33]. Findings relating to the effect of gender on survival have been inconsistent, with a significant protective effect for males observed more frequently in retrospective studies compared to prospective population-based studies [3, 26, 34–36]. In our study, the trends for older age of onset and female gender did not reach statistical significance, and inclusion of these of variables in our multivariate model made no difference to the overall results (data not shown).

Genetic data, though available, was not used in our prognostic model because our aim was to incorporate variables that can be obtained on first patient encounter. Reviewing the risk group distribution among patients carrying the *C9orf72* pathogenic hexanucleotide repeat in both populations revealed that this mutation was rare among patients categorised as Low risk. This is consistent with previous reports from our group and other groups suggesting worse prognoses in this group and it suggests the patients with atypical long survival times are likely to harbour either no mutation or a new yet to be identified gene mutation.

The limitations of this study include its retrospective nature. Although the data were collected prospectively, the design of the study included extensive cognitive testing, and did not prioritise body mass index or forced vital capacity, as many of the patient assessments were conducted in patients’ homes to maximise recruitment. In addition, patients with particularly aggressive disease are less likely to be recruited to cognitive studies. However, this effect is unlikely to be large in this study as the cohorts from both centres were population-based and data were gathered by home-visits in both studies.

In conclusion, we have shown that information gathered on first patent visit can be used to reliably predict prognosis. This prognostic algorithm is more reliable than that predicted by ALSFRS slope alone, and is sufficiently simple to enable its use by clinicians in busy clinics for individual patient prognostication. In addition, the simplicity and reliability of the model has the potential to improve stratification protocols in future clinical trials. Notwithstanding the fact that we have validated the algorithm in two populations, prospective studies replicating our findings using brief cognitive screening tools such as the ECAS are also desirable.
